# Role of the Nitric Oxide Reductase NorVW in the Survival and Virulence of Enterohaemorrhagic *Escherichia coli* during Infection

**DOI:** 10.3390/pathogens9090683

**Published:** 2020-08-21

**Authors:** Marion Gardette, Julien Daniel, Estelle Loukiadis, Grégory Jubelin

**Affiliations:** 1 Université Clermont Auvergne, INRAE, MEDIS UMR454, F-63000 Clermont-Ferrand, France; gardette.marion@gmail.com (M.G.); julien.daniel@inrae.fr (J.D.); 2 Université de Lyon, CNRS, INRAE, Université Claude Bernard Lyon 1, VetAgro Sup, Laboratoire d’Ecologie Microbienne, F-63280 Marcy l’Etoile, France; estelle.loukiadis@vetagro-sup.fr; 3 VetAgro Sup, Laboratoire national de référence des E. coli, F-63280 Marcy-l’Etoile, France

**Keywords:** EHEC, Shiga toxin, virulence, nitric oxide, NO reductase

## Abstract

Enterohaemorrhagic *Escherichia coli* (EHEC) are bacterial pathogens responsible for life-threatening diseases in humans, such as hemolytic and uremic syndrome. It has been previously demonstrated that the interplay between EHEC and nitric oxide (NO), a mediator of the host immune innate response, is critical for infection outcome, since NO affects both Shiga toxin (Stx) production and adhesion to enterocytes. In this study, we investigated the role of the NO reductase NorVW in the virulence and fitness of two EHEC strains in a murine model of infection. We determined that the deletion of *norVW* in the strain O91:H21 B2F1 has no impact on its virulence, whereas it reduces the ability of the strain O157:H7 620 to persist in the mouse gut and to produce Stx. We also revealed that the fitness defect of strain 620 Δ*norVW* is strongly attenuated when mice are treated with an NO synthase inhibitor. Altogether, these results demonstrate that the NO reductase NorVW participates in EHEC resistance against NO produced by the host and promotes virulence through the modulation of Stx synthesis. The contribution of NorVW in the EHEC infectious process is, however, strain-dependent and suggests that the EHEC response to nitrosative stress is complex and multifactorial.

## 1. Introduction

Enterohaemorrhagic *Escherichia coli* (EHEC) are foodborne pathogens responsible for intestinal disorders in humans that can evolve into life-threatening diseases, such as hemorrhagic colitis and hemolytic-uremic syndrome (HUS) [1,2,3]. EHEC are classified by O:H serotype based on their lipopolysaccharide and flagellar antigens. Among the limited number of serotypes frequently involved in human infections [4], O157:H7 is recognized as the major serotype involved in EHEC outbreaks and sporadic cases of infection worldwide [5]. However, the incidence of non-O157:H7 strains has steadily increased these last years, and serotypes such as O26:H11, O103:H2, O91:H21, and O80:H2 are now involved in a substantial proportion of infection cases [6,7]. The cardinal virulence factor in EHEC is Shiga toxins (Stx), which are AB_5_ proteins where the A subunit is the catalytic protein and the five B subunits are required for binding at the surface of target cells [8]. Once produced in the gut, Stx crosses the intestinal epithelial barrier and gains access to the systemic circulation, where it targets the glycolipid globotriaosylceramide-3 (Gb3) receptors of endothelial cells [9]. The internalization of Stx alters the ribosomal function and induces the necrosis or apoptosis of vascular endothelial cells, leading to symptoms such as hemorrhagic colitis and HUS [10]. EHEC can produce two distinct forms of Stx—Stx1 and Stx2—alone or in combination, and several subtypes have been described for each type of Stx [11]. The *stx1* and *stx2* genes are located within lambdoid phages integrated within the bacterial chromosome, and their expression is mainly activated through the induction of the SOS response [12]. Epidemiological studies have shown that EHEC strains producing Stx2, especially subtypes Stx2a and Stx2d, are more commonly associated with serious human diseases such as HUS than Stx1-producing strains [13,14].

Although critical for EHEC virulence, Stx is not sufficient per se to cause disease in humans, and many Shiga-toxin-producing *E. coli* (STEC) strains have never been associated with cases of infection [4]. The virulence potential of EHEC strains results from the association of *stx* genes with other virulence or fitness factors encoding genes located on the chromosome or on mobile elements such as plasmids. Among these factors, the Locus of Enterocyte Effacement (LEE) is frequently detected in EHEC strains, causing severe infections. LEE genes encode the type III secretion system (T3SS) which is involved in the formation of attaching and effacing (A/E) lesions on infected epithelial cells, leading to an intimate attachment of EHEC to the gut mucosa [15]. The *norV* gene was also identified as a putative virulence determinant in the EHEC O157:H7 strain TW14359, which has been involved in an outbreak with a particularly high rate of HUS [16,17]. *norV* and the co-transcribed *norW* gene encode a flavorubredoxin and a NADH:(flavo)rubredoxin reductase, respectively, that are involved in nitric oxide (NO) detoxification under anaerobic and microaerobic growth conditions [18,19,20]. NO is a highly reactive inorganic free radical with pleiotropic functions in mammals and has notably antimicrobial properties that make it efficient in fighting against incoming pathogens as part of the host immune response [21]. In the case of EHEC, the interplay between NO and the pathogen appears to be even more critical for the outcome of infection, since NO has been shown to modulate the expression of both the *stx* and LEE genes [22,23,24,25]. In addition, NorV has been shown to promote EHEC survival and Stx2 production within macrophages, suggesting that NorV contributes to EHEC virulence [26].

In this context, the aim of this work was to evaluate the role of *norV* in the pathogenicity of two EHEC strains of serotype O157:H7 or O91:H21, using mice for an animal model of infection. We determined that the deletion of *norVW* highly affects the efficiency of the strain O157:H7 to colonize the gut and release Stx in vivo. In contrast, *norVW* appears to be dispensable for the virulence of the O91:H21 strain. We also demonstrated that *norVW* is critical for the efficient fitness of the O157:H7 strain in the gut of infected mice. Importantly, the advantage procured by the presence of *norVW* is significantly decreased when NO production by the host is inhibited, strongly suggesting that NorV is involved in NO detoxification during the infectious process. Altogether, our work highlights the role of the NO reductase NorV in the fitness and virulence of some, but not all, EHEC strains during the infectious process.

## 2. Results

### 2.1. Selection of norV^+^ EHEC Strains

Two forms of the *norV* gene exist in EHEC, an intact and functional gene (*norV^+^*) and an inactive gene with a 204 bp deletion (*norV^−^*) [19,26]. We determined the *norV* status in 34 EHEC strains belonging to different serotypes frequently involved in human infection (Table 1). Three of the six tested O157:H7 isolates and all of the non-O157 strains possess an intact *norV* gene. No correlation was observed between the *norV* status and the presence of virulence genes *eae* (encoding intimin), *stx1*, and *stx2*. These data are consistent with previous results, indicating that the truncated form of the *norV* gene is strictly detected in the O157:H7 strains [19,26]. Three O157:H7 and two non-O157:H7 strains carrying the *norV^+^* gene were next selected for further investigation. For the O157:H7 serotype, the selected strains were (i) FCH6, a typical O157 strain producing the Stx2a variant and involved in an outbreak ([27] and personal communication with Sergentet D. from [28]); (ii) 620, a sorbitol-fermenting clone producing Stx2a and associated with a high incidence of HUS and high fatality rate (personal communication with Loukiadis E. from [29]); (iii) 540, a Stx1 and Stx2a-producing strain which has been involved in sporadic cases of infection ([30] and personal communication with Loukiadis E. from [29]). The three O157:H7 strains all possess the LEE operon. The two selected non-O157:H7 strains were 21765, an LEE^+^ and Stx2a^+^ O26:H11 strain [31], and B2F1, a LEE^−^ O91:H21 strain producing two Stx2d variants [32]. Both isolates were recovered from HUS cases [33,34].

### 2.2. Virulence of norV^+^ EHEC Strains in a Mouse Model of Infection

**Figure 1:**
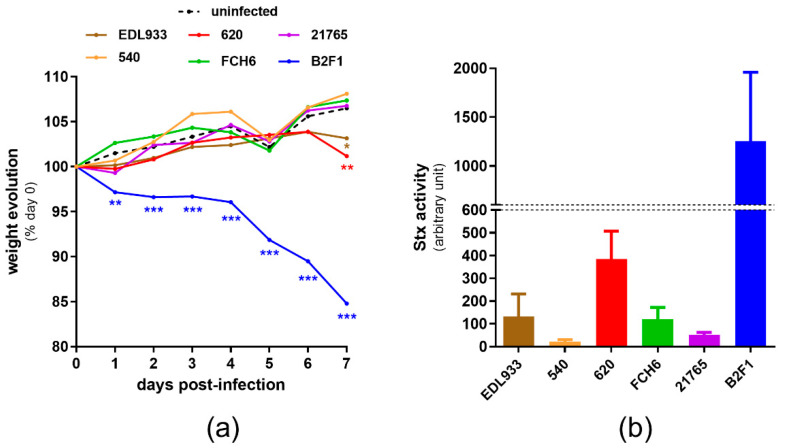
Virulence potential of EHEC strains in mice. (**a**) Groups of 5 to 20 mice were infected by indicated strains at day 0, and the mouse weights were determined daily over a 7-day period. Data are presented as the percentage relative to animal weight at the day of infection, (day 0) and curves represent mean values. A multiple two-tailed unpaired t-test was applied to compare each infected group with the uninfected group every day. * *p* < 0.05, ** *p* < 0.01, *** *p* < 0.001. (**b**) At day 3 post-infection, the Stx activity of fecal samples was quantified using the Vero-d2EGFP cell line. Results are presented as means and standard deviations, with at least 4 mice per group.

We first determined the virulence potential of the selected strains using a mouse model of infection (see M&M). Streptomycin-treated mice were orally infected with a streptomycin-resistant variant of each strain (hereafter named wild-type (WT) strains for more convenience), as well as with the reference strain EDL933 (an O157:H7 strain with a truncated form of *norV*), and body weight was recorded daily to evaluate the severity of infection (Figure 1a). Mice infected with 540, FCH6, or 21,765 strains gained body weight during the course of infection, similarly to uninfected mice. Infection with EDL933 and 620 led to the same body weight pattern, characterized by a regular increase during the first 6 days and a slight but significant decrease at day 7 post-infection.

In contrast, the B2F1-infected mice lost weight as soon as day 1 post-infection, and their body weight was significantly lower than that of uninfected mice during the entire course of infection. In addition, the B2F1-infected mice developed serious clinical signs of disease, as previously described [35], and 30% of the mice were moribund or died during the experiment (Figure S1). We also evaluated the amount of released Stx at day 3 post-infection through the quantification of the Stx activity from fecal samples using Vero-d2GFP, a green fluorescent protein (GFP)-producing cell line used to monitor protein synthesis inhibition [36]. These data revealed that B2F1 and 620 present the highest level of Stx activity in vivo among the tested strains (Figure 1b), correlating well with the degree of weight loss and symptoms observed in the infected mice. B2F1 and 620 strains were then used to investigate the role of the *norVW* operon in EHEC virulence and fitness.

### 2.3. Role of the norVW Operon in EHEC Virulence

**Figure 2:**
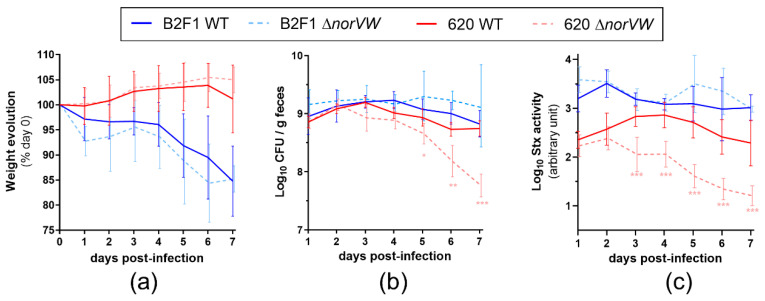
Deletion of the *norVW* operon differentially affects the virulence of strains 620 and B2F1. Groups of 10 to 20 mice were infected with WT or Δ*norVW* mutant from the strains 620 or B2F1. (**a**) Mouse weights were determined daily following infection, and body weight curves are presented as the percentage relative to animal weight at the day of infection (day 0). Curves represent mean values with standard deviation (SD). (**b**) At the indicated time points, EHEC shedding was quantified by plating fecal samples on LB + Sm plates. Curves represent mean values with SD. (**c**) Stx activity from fecal samples was quantified using the Vero-d2EGFP cells. Curves represent mean values with SD. A multiple two-tailed unpaired t-test was performed to compare the WT and Δ*norVW* groups of mice infected with 620 or B2F1 every day. * *p* < 0.05, ** *p* < 0.01, *** *p* < 0.001.

To assess the contribution of *norVW* to EHEC virulence, we orally inoculated mice with WT or Δ*norVW* mutant from 620 or B2F1 strains and evaluated the infection outcome by analyzing the weight loss and clinical signs of the animals. The body weight curves were not significantly different between the WT and Δ*norVW* strains both for 620 and B2F1 (Figure 2a). Indeed, the mice infected with 620 WT or Δ*norVW* showed a weight increase during the first 6 days post-infection, whereas the mice challenged with B2F1 WT or Δ*norVW* both exhibited serious clinical symptoms and considerable weight loss.

We next evaluated the consequence of *norVW* deletion on the ability of strains to persist in the gut. In mice infected with B2F1, the fecal shedding was stable during the course of infection, both for the WT and Δ*norVW* strains (Figure 2b). In contrast, significantly fewer CFU were recovered in the feces of mice infected with 620 Δ*norVW* than from those infected with 620 WT from day 5 to day 7 post-infection (Figure 2b). This result suggests that *norVW* contributes to the persistence of strain 620 in the gut lumen. To assess whether *norVW* deletion also affects the ability of EHEC to adhere to the gut epithelium in vivo, adherent bacteria were quantified from colonic tissues at day 7 post-infection (Figure S2). The number of adherent EHEC was highly variable between the mice, and we were not able to detect significant differences between the WT and Δ*norVW* strains from both the 620 and B2F1 isolates. We next investigated whether *norVW* deletion affects the activity of Stx in vivo via the quantification of Stx activity from animal feces (Figure 2c). First, we noticed that the Stx activities recorded from the B2F1-infected mice were higher than those recorded from animals infected with 620 during the entire course of infection, correlating well with the severity of symptoms observed for these mice. We next compared the Stx activities between the WT and Δ*norVW* mutant for each strain. We did not observe differences between the mice infected with B2F1 WT or mutant strains, demonstrating that the *norVW* operon did not alter the Stx activity in B2F1. In contrast, the Stx activities recorded from 620 Δ*norVW*-infected mice were significantly lower than the Stx activities measured from mice infected with the WT strain between day 3 and day 7 post-infection. This data reveals that the deletion of the *norVW* operon in 620 negatively affects the level of Stx activity in infected animals. Altogether, these data demonstrate that the virulence of the Δ*norVW* mutant is attenuated for strain 620, but not for strain B2F1, and highlight that the expression of *norVW* can influence the virulence of some, but not all, EHEC strains.

### 2.4. Role of the norVW Operon in the Fitness of EHEC during Mouse Infection and Influence of NO Synthase Activity from the Host

**Figure 3:**
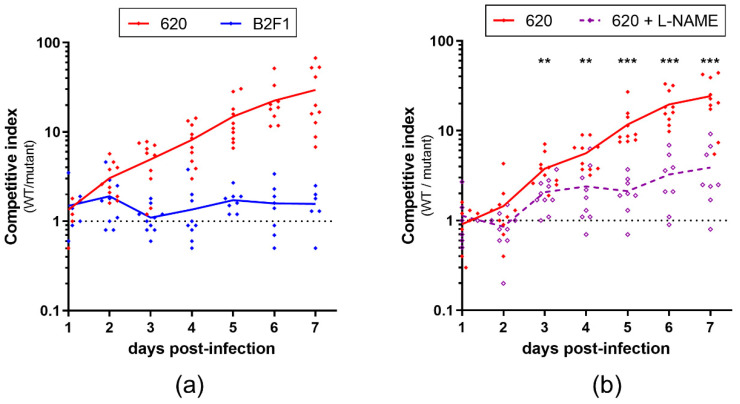
Role of the *norVW* operon in EHEC fitness during the infection and influence of NO synthase activity from the host. (**a**) Mice were co-infected with an equal mixture of WT and Δ*norVW* mutant from strains 620 or B2F1. At the indicated time points, feces were sampled and spotted on LB + Sm plates and LB + Sm + Kan plates to count, respectively, the WT + Δ*norVW* mutants and Δ*norVW* mutants alone. The WT population was obtained by subtracting the Δ*norVW* mutant colony-forming unit (CFU) from the total EHEC CFU. Competitive indices (ratio WT/mutant) were calculated for each animal. Each dot represents one mouse, and curves represent mean values. (**b**) A similar competition assay was performed using the 620 WT and Δ*norVW* strains, and mice from one group were treated with the NOS inhibitor L-NAME throughout the experiment and mice from the other group were left untreated. A multiple two-tailed unpaired t-test was performed to compare both groups every day. ** *p* < 0.01; *** *p* < 0.001.

To assess the contribution of *norVW* in the fitness of strains 620 and B2F1 in the gut, the mice were co-infected with an equal ratio of WT and Δ*norVW* strains, and both populations were monitored over the course of infection to determine the competitive indices (ratio WT/mutant) (Figure 3a).

**Figure 4:**
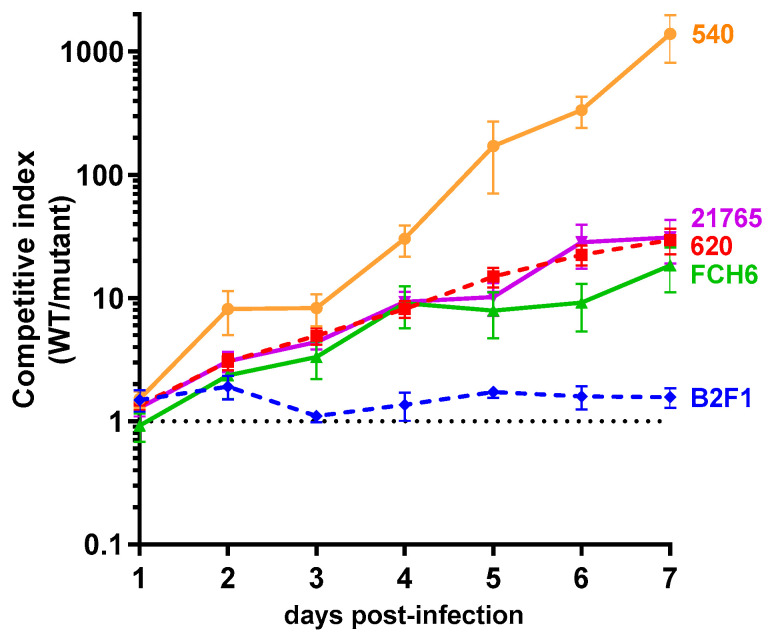
Role of the *norVW* operon in the fitness of other EHEC strains during infection. Groups of 10 mice were co-infected with an equal mixture of WT and Δ*norVW* mutant from strains FCH6, 540, and 21,765 (and 620 and B2F1 as references). At different time points, feces were sampled and spotted on LB + Sm plates and LB + Sm + Kan plates to count, respectively, the WT + Δ*norVW* mutants and Δ*norVW* mutants alone. The WT population was obtained by subtracting the Δ*norVW* mutant CFU from the total EHEC CFU. Competitive indices (ratio WT/mutant) were calculated for each animal. Curves represent the mean values with standard errors of the mean.

The deletion of genes *norVW* had no impact on the B2F1 colonization efficiency, since the calculated competitive indices remained close to 1 throughout the experiment. In contrast, the competitive indices greatly increased for 620 and reached a mean value of 30 at day 7 post-infection, indicating that 620 WT outcompetes the Δ*norVW* mutant in vivo. These data demonstrate that the *norVW* operon contributes to the fitness of strain 620 in the mouse gut but does not influence the behavior of strain B2F1. Because NO is released in the mouse gut during EHEC infection [25], we wondered if the fitness defect observed with the 620 Δ*norVW* strain is related to the NO production by the host during infection. The mice were then co-infected with the 620 WT and Δ*norVW* strains and treated or not with L-NAME, a specific inhibitor of NO synthase (NOS) activity (Figure 3b). The competitive indices calculated from the mice treated with L-NAME stayed very low (maximal mean value of 3.9 at day 7 post-infection), and were significantly lower than those obtained from the untreated mice. These differences were observed as soon as day 3 post-infection and increased over time until the end of the experiment. These data demonstrate that the competitive advantage of the WT strain over the Δ*norVW* mutant in the mouse gut is due, at least partially, to NO production by the host. Finally, we also evaluated the role of NorVW in the fitness of the three other EHEC strains initially selected (540, FCH6, and 21765) by co-infection experiments (Figure 4). For the three strains, the mutant was outcompeted by the WT strain by a factor ranging from 19 to 1400 at day 7 post-infection. These data indicate that NorVW also participates in the fitness of other O157 and non-O157 strains during mouse infection, as initially determined with the strain 620.

## 3. Discussion

During the past decades, NO has been recognized as an important player in the immune system, participating notably to host defense against infectious agents [21]. In the case of EHEC infection, several works also demonstrated that the pathogen is able to sense NO in the gut and to adapt its gene expression accordingly, making the interplay between NO and EHEC critical for the infection outcome [22,25,37,38,39]. Among the NO-regulated factors, the NO reductase NorVW was identified as a putative virulence determinant in the EHEC O157:H7 strain TW14359 and was shown to promote EHEC survival within macrophages [16,26]. NorV was also identified as one of the immunogenic antigens expressed by EHEC within the gastrointestinal tract of HUS patients [38]. The aim of this study was to examine the contribution of the NO reductase NorVW to EHEC pathogenesis. Using a mouse model of infection, we demonstrated that the deletion of the gene *norVW* impacts the virulence of strain O157:H7 620, but not of strain O91:H21 B2F1, suggesting that the activity of the NO reductase is important in the infectious process of some, but not all, EHEC strains. In addition, the fitness of four of the five tested EHEC strains was affected in the gut of infected mice in the absence of the *norVW* operon. The altered fitness of 620 Δ*norVW* was furthermore attenuated in L-NAME-treated mice, producing a lower amount of NO. In this condition, the fitness of the WT and Δ*norVW* strains was nearly equivalent, and this data strongly suggests that NorVW plays a role in the detoxification of NO produced by the host in response to infection [25]. In addition to NorVW, *E. coli* harbor three other enzymes known to detoxify NO: the flavohaemoglobin Hmp, which oxidizes NO to nitrate or reduces NO to nitrous oxide [19,40]; the periplasmic nitrite reductase NrfA, which transforms NO into ammonia [41]; and the hybrid cluster protein Hcp, which catalyzes the reduction of NO to nitrous oxide [42]. In bacterial cells, the quantitative contribution of Hmp, NrfA, Hcp, and NorV to NO detoxification varies significantly depending on the NO and dioxygen concentrations, since both molecules affect the synthesis and/or activity levels of these enzymes [43,44,45]. However, the variations in the cooperativity level of NO-detoxifying enzymes have never been examined between *E. coli* strains. Whereas genes *hmp*, *nrfA*, *hcp*, and *norVW* belong to the core genome of *E. coli*, it is conceivable that their expression level and/or enzyme efficiency differ between strains 620 and B2F1, explaining why NorVW is important for the fitness and virulence of 620 but not of B2F1. Otherwise, B2F1 may also possess other unidentified NO scavenging mechanisms, as was shown in the uropathogenic *E. coli* strain CFT073 [46].

One important feature observed in our study is that *norVW* deletion leads to a significant decrease in the Stx activity level in the gut of mice infected by 620, but not of mice infected by B2F1. It should be noticed that 620 and B2F1 produce distinct variants of Stx2 (Stx2a for 620 and two Stx2d for B2F1), and it is conceivable that their regulation may differ. In the reference strain EDL933, which produces the Stx2a variant like 620, NO modulates the *stx* gene expression in a RecA-dependant manner by affecting the induction level of the phage lytic cycle [39]. Interestingly, it has been shown that the regulation of the two *stx2*d operons in B2F1 is different, and only one *stx* allele has an expression linked to its bacteriophage induction [32], leaving open the possibility that the RecA-dependent effect of NO may not occur for one of the *stx* alleles. The reduction in the Stx activity observed in mice infected by 620 Δ*norVW* occurred as soon as day 3 post-infection and did not result from a reduced level of gut colonization by the mutant, which was not observed before day 5 post-infection (compare Figure 2b,c). Actually, the reduced colonization ability of 620Δ*norVW* could even be a consequence of the decreased Stx activity, since it was previously reported that Stx may enhance the capacity of EHEC O157:H7 to adhere to epithelial cells and to colonize the mouse intestine [47,48]. The low Stx activity recorded from mice infected with the Δ*norVW* mutant probably results from an increase in the NO concentration in the absence of NO reductase, and suggests that NO represses Stx production. The influence of NO on Stx synthesis has been well demonstrated in several studies, but appears to be highly variable. Indeed, NO was shown in vitro to inhibit Stx production during standard LB culture [39], whereas it activates Stx synthesis under anaerobic conditions [24]. The infection of macrophages with *norV*^+^ EHEC strains reduces the NO concentration and leads to a higher level of Stx2 than within macrophages infected with *norV* mutants [26]. Our team also recently demonstrated that the treatment of EHEC-infected mice with a NO synthase inhibitor significantly decreases the Stx activity in the gut [25]. Altogether, these data reveal that the modulation of Stx synthesis by NO (and indirectly by the activity of NO detoxification systems) is highly complex and sensitive, and affected by multiple environmental conditions.

The role of NO detoxification systems in virulence has been highlighted in vivo for other bacterial pathogens. If numerous studies have demonstrated the role of Hmp in the virulence of *Salmonella enterica*, *Vibrio cholera*, *Staphylococcus aureus*, *Yersinia pestis*, and uropathogenic *E. coli* [49,50,51,52,53,54], very few data exist for the less studied NO detoxification systems. In the case of NorVW, it has been shown that gene deletion affects the virulence of *Pseudomonas aeruginosa* in a silkworm model [55] and the virulence of *Aeromonas hydrophila* in Zebrafish, a bacterium responsible for diseases in amphibians, fish, and reptiles [56]. In contrast, *norV* deletion in *S. enterica* did not affect the virulence in mice [49,57]. To our knowledge, the contribution of NorV in the infectious process of intestinal or extra-intestinal pathogenic *E. coli* has never been documented, and this study is the first to demonstrate a role of the NO reductase NorV in the virulence of *E. coli*. Intriguingly, the occurrence of a deletion event in *norV* gene during the phylogenetic evolution of O157 is contradictory with the role of NorV in EHEC virulence. The truncated form of *norV* is, however, essentially detected in Stx1^+^ Stx2^+^ EHEC strains and very rarely in EHEC strains, producing only Stx2 [58]. Because the latter group is more commonly associated with the development of severe symptoms in infected patients [59,60] and the molecular reasons for this are still unknown, it is tempting to speculate that the presence of a functional NO reductase NorV in these strains can contribute to their high potential for virulence. Further investigations are required to understand and characterize the contribution of NorV and other NO detoxification systems in the virulence of EHEC, potentially leading to their exploitation as new targets for antibacterial agents.

## 4. Materials and Methods

### 4.1. Bacterial Strains, Growth Conditions, and Construction of Mutants

The bacterial strains and plasmids used in this study are listed in Table S1, and the primer sequences are listed in Table S2. The status of the *norV* gene was determined in each EHEC strain either by DNA sequence analysis if the genome was publicly available or by PCR using the primers norV^+^-F and norV^+^-R. For selected strains (620, FCH6, 540, 21,765, and B2F1), a streptomycin-resistant variant was constructed by replacing the *rspL* gene with gene *rpsL150* amplified from MC4100 (confers resistance to streptomycin), as described in [61]. The deletion of the *norVW* operon was performed in EHEC strains using the one-step PCR-based method [62] using the primers norVW-mut-F and norVW-mut-R. Chromosomal deletions were confirmed by PCR using the primers norVW-verif-F and norVW-verif-R, followed by DNA sequencing. Bacteria were routinely grown in Luria–Bertani (LB) medium (Sigma-Aldrich, St Quentin Fallavier, France) at 37 °C, unless otherwise indicated. When required, antibiotics were used at the following concentrations: ampicillin (Amp), 50 µg mL^−1^; kanamycin (Kan), 25 µg mL^−1^; atreptomycin (Sm), 50 µg mL^−1^.

### 4.2. Mouse Infection

C57BL/6 mice with specific-pathogen-free (SPF) status were purchased from Janvier Labs (Le-Genest-St-Isle, France). Female mice aged 5 weeks were used throughout the experiments. They were housed in cages containing no more than five animals, maintained under a 12 h light/dark cycle at a temperature of 21 ± 2 °C, and fed with standard diet and water ad libitum. The experiments performed herein were reviewed and approved by the Auvergne Committee for Animal Experimentation C2EA (Agreement N°7289-2016093010075533). Mouse experiments were performed with 5–20 mice per group. The mice were given drinking water containing 5 g/L of streptomycin sulfate (Sigma-Aldrich, St Quentin Fallavier, France) one day prior to infection and during the 7 days following infection. For some groups, drinking water was also supplemented with 1 g/L of the NOS inhibitor N^ω^-nitro-L-arginine methyl ester hydrochloride (L-NAME; Enzo Life Science, Villeurbanne, France), and changed daily over the course of the experiment. The mice were infected intragastrically at day 0 with 100 µL of PBS (Sigma-Aldrich, St Quentin Fallavier, France) containing 10^7^ bacteria. For this, the bacteria were grown in LB to the mid-logarithmic phase, collected by centrifugation, and resuspended to a concentration of 10^8^ CFU/mL in sterile phosphate-buffered saline (PBS). Uninfected mice were given PBS only. The body weight and clinical signs of mice were monitored daily to evaluate the severity of infection. Mice presenting a weight loss >15% compared to their body weight at day 0, or presenting severe clinical symptoms such as ataxia and lethargy, were immediately euthanized. Fecal samples were collected every day following infection. Feces were homogenized in PBS and diluted before plating on LB + Sm. Resuspended feces were centrifuged, and the supernatant was filtered and stored at −80 °C for subsequent Stx activity quantification. The mice were euthanized at day 7 post-infection. At necropsy, a piece of colon was collected, cut longitudinally, and washed extensively in PBS. Crushing in PBS, dilution, and plating were performed to quantify the bacteria adhering to the gut mucosa.

For the co-infection experiments, the mice were infected with a mix containing 10^7^ each of the WT and Δ*norVW* strains. The bacteria were enumerated in feces during the 7 days post-infection. Fecal samples dilutions were plated on LB + Sm or LB + Sm + Km agar plates to count the WT + Δ*norVW* strains and Δ*norVW* alone, respectively. Competitive indices were calculated daily by dividing the output ratio (WT/mutant) by the corresponding input ratio (WT/mutant from mouse inoculum).

### 4.3. Stx activity Quantification

The Shiga toxin activity in the fecal samples was measured using a Vero-d2EGFP cell line that harbors a destabilized variant (half − life = 2 h) of enhanced green fluorescent protein (EGFP), as described previously [36,63]. Purified Stx2 (Toxin Technology, Sarasota, FL, USA) was used as an internal standard. Three days prior to intoxication, the Vero-d2EGFP cells were seeded in black 96-well plates with a clear bottom at 3 × 10^4^ cells per well. They were grown in 5% CO_2_ at 37 °C under humidified conditions in a complete medium made of Annonces

Dulbecco′s modified Eagle′s medium (DMEM) supplemented with 10% fetal bovine serum (Gibco, Fisher Scientific, Illkirch, France), Zell Shield (Minerva Biolabs, Berlin, Germany), and 200 µg mL^−1^ of Geneticin (Gibco, Fisher Scientific, Illkirch, France). The samples to be tested as well as purified with Stx2 were diluted in complete medium, transferred to Vero-d2EGFP-containing plates, and incubated at 37 °C for 16 h in a 5% CO_2_ humidified incubator. After incubation, the samples were removed and 100 µL of PBS was added to each well. The EGFP fluorescence from the Vero-d2EGFP was quantified in a Spark microplate reader (Tecan, Lyon, France) with an excitation at 485 ± 20 nm and emission at 530 ± 20 nm. The Stx activity was expressed as an arbitrary unit by comparing the fluorescence values from the standard curve obtained with purified Stx2.

### 4.4. Statistical Analyses

All the statistical analyses were performed using the Prism software version 8 (GraphPad Software, San Diego, California, USA). Each dataset was analyzed by the ROUT method to exclude outliers with a Q value of 1%. An unpaired two-tailed Student’s t test was used to determine significant differences between the two groups. *p* < 0.05 was considered significant.

## Figures and Tables

**Table 1:** Distribution of *norV*, *eae*, and *stx* genes in 34 enterohaemorrhagic *Escherichia coli* (EHEC) strains ^a^.

Serotype	Strain	*norV*	*eae* ^b^	*stx1*	*stx2*
O157:H7	620	+	+	–	+
	540	+	+	+	+
	FCH6	+	+	–	+
	RD9	-	+	+	+
	EDL 933	-	+	+	+
	Sakaï	-	+	+	+
O26:H11	21765	+	+	–	+
	37.40	+	+	+	–
	279/8	+	+	+	–
	11368	+	+	+	–
O103:H2	PMK5	+	+	+	–
	590	+	+	+	–
	2503	+	+	+	–
	03.35	+	+	+	–
	2455-1	+	+	+	+
O111:H8	CL37	+	+	–	–
	J43	+	+	+	–
	622-4	+	+	+	+
O145:H28	2513-21	+	+	–	+
	991	+	+	+	–
	1036	+	+	–	+
O121:H19	12652	+	+	–	+
	12805	+	+	–	+
	S3075	+	+	–	+
O45:H2	12047	+	+	+	–
O91:H21	13199	+	–	+	+
	B2F1	+	–	–	+
	13694	+	–	–	+
O113:H21	13341	+	–	–	+
	14032	+	–	–	+
O113:H4	13137	+	–	+	–
O80:H2	38009	+	+	–	+
	RDEx444	+	+	–	+
	40963	+	+	–	+

^a^ The symbols + and – indicate, respectively, the presence and the absence of indicated genes except for *norV*, for which the symbol - indicates the presence of a truncated form of the gene. ^b^ *eae* encodes intimin and is a marker of the presence of the locus of enterocyte effacement (LEE).

## Supplementary Materials

The following are available online at https://www.mdpi.com/2076-0817/9/9/683/s1. Figure S1. Survival rate of mice infected with EHEC strains. Figure S2. Deletion of the *norVW* operon does not affect EHEC adhesion to colonic mucosa. Table S1. Bacterial strains and plasmids used in this study. Table S2. Primers used in this study. References [64,65,66,67,68,69,70] are cited in the Supplementary Materials.
