# Supplementary material for: Role of the Nitric Oxide Reductase NorVW in the Survival and Virulence of Enterohaemorrhagic Escherichia coli during Infection
Source: Pathogens. 2020 Aug 21;9(9):683. doi: 10.3390/pathogens9090683 (PMC7558590; doi:10.3390/pathogens9090683)
Supplement: Supplementary file 1 [file pathogens-09-00683-s001.pdf]

pathogens

MDPI

Supplementary Data

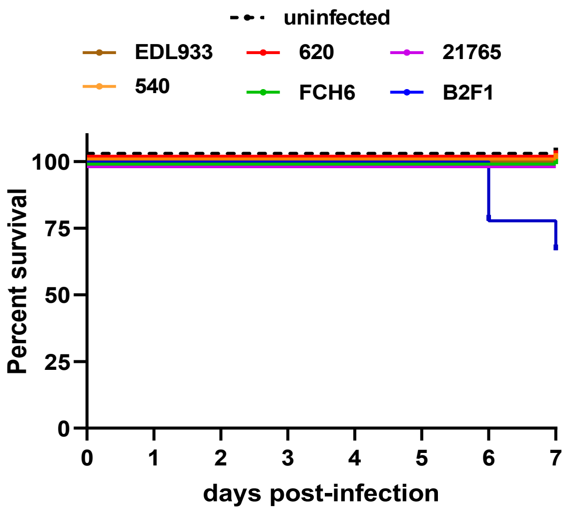

Figure S1. Survival rate of mice infected with EHEC strains. The survival time and rate were recorded for 7 days after infection (day 0) with the indicated EHEC strains.

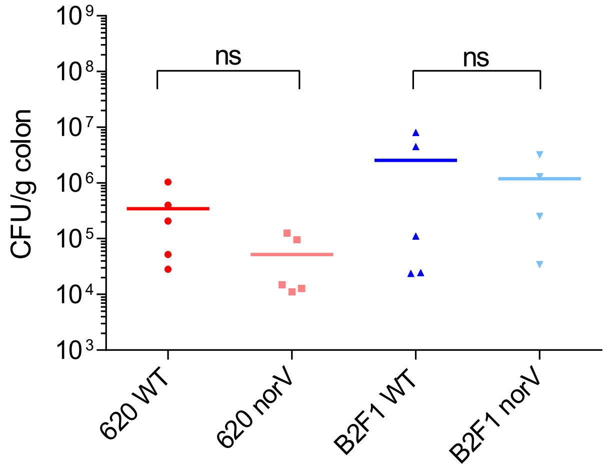

Figure S2. Deletion of the *norVW* operon does not affect EHEC adhesion to colonic mucosa. At day 7 post-infection, mice were euthanized and the colon were sampled, washed in PBS, crushed and then plated on LB + Sm plates in order to quantify mucosa-associated EHEC. Each dot represents one mouse and means are indicated as a line. A two-tailed unpaired t-test was applied to compare both groups. ns: not significant.

Table S1. Bacterial strains and plasmids used in this study.

Strains or Plasmids	Description	References
Bacterial Strains		
620 WT	O157:H7 isolated from bovine meat and implicated in human infection	[29]
620 Sm^R	Streptomycin-resistant derivative of 620; Sm^R	This study
620 Δ*norVW*	620 Sm^R Δ*norVW*; Sm^R Kan^R	This study
B2F1	O91:H21 isolated from human feces	[33]
B2F1 Sm^R	Streptomycin-resistant derivative of B2F1; Sm^R	This study
B2F1 Δ*norVW*	B2F1 Sm^R Δ*norVW*; Sm^R Kan^R	This study
FCH6	O157:H7 isolated from raw goat's milk cheese and implicated in human infection	[27]
FCH6 Sm^R	Streptomycin-resistant derivative of FCH6; Sm^R	This study
FCH6 Δ*norVW*	FCH6 Sm^R Δ*norVW*; Sm^R Kan^R	This study
540 WT	O157:H7 isolated from bovine meat and implicated in human infection	[30]
540 Sm^R	Streptomycin-resistant derivative of 540; Sm^R	This study
540 Δ*norVW*	540 Sm^R Δ*norVW*; Sm^R Kan^R	This study
21765	O26:H11 isolated from raw goat's milk cheese and implicated in human infection	[34]
21765 Sm^R	Streptomycin-resistant derivative of 21765; Sm^R	This study
21765 Δ*norVW*	21765 Sm^R Δ*norVW*; Sm^R Kan^R	This study
EDL933 Sm^R	Streptomycin-resistant derivative of O157:H7 EDL933; Sm^R	[37]
RD9	O157:H7 isolated from bovine meat and implicated in human infection	[64]
Sakaï	O157:H7 isolated from human feces	[65]
37.40	O26:H11 isolated from bovine meat and implicated in human infection	[66]
279/8	O26:H11 isolated from raw cow's milk cheese and implicated in human infection	[30]
11368	O26:H11 isolated from human feces	[67]
PMK5	O103:H2 isolated from human feces	[68]
590	O103:H2 isolated from raw cow's milk cheese and implicated in human infection	[30]
2503	O103:H2 isolated from raw cow's milk cheese and implicated in human infection	[30]
03.35	O103:H2 isolated from bovine meat and implicated in human infection	[30]
2455-1	O103:H2 isolated from raw cow's milk cheese and implicated in human infection	[30]
CL37	O111:H8 isolated from human feces	[69]
J43	O111:H8 isolated from bovine feces	[70]
622-4	O111:H8 isolated from raw cow's milk cheese and implicated in human infection	[30]
2513-21	O145:H28 isolated from raw cow's milk cheese and implicated in human infection	[30]
991	O145:H28 isolated from bovine meat and implicated in human infection	[30]
1036	O145:H28 isolated from raw cow's milk cheese and implicated in human infection	[30]
12652	O121:H19 isolated from human feces	[30]
12805	O121:H19 isolated from human feces	[30]
S3075	O121:H19 isolated from human feces	[30]
12047	O45:H2 isolated from human feces	[30]
13199	O91:H21 isolated from bovine meat and implicated in human infection	[30]
13694	O91:H21 isolated from raw cow's milk cheese and implicated in human infection	[30]
13341	O113:H21 isolated from raw cow's milk cheese and implicated in human infection	[30]
14032	O113:H21 isolated from bovine meat and implicated in human infection	[30]
13137	O113:H4 isolated from sheep meat and implicated in human infection	[30]
Plasmids		
pKD46	Temperature-sensitive (replicates at 30°C) plasmid bearing genes encoding the Red recombinase, Amp^R	[62]
pKD4	Template plasmid for amplification of a kanamycin cassette, Amp^R, Km^R	[62]

Table S2. Primers used in this study.

Name	Sequence 5′-3′	References
norV⁺-F	GGCCATGGAACACGCTGAATAAATTG	[58]
norV⁺-R	CCAAGCTTTTGCCTCCGATGCCA	[58]
rpsl-F	CTGACCAATGACGCGACGAC	This study
rpsl-R	GACTGGTCAAATTTCGAACGA	This study
norVW-mut-F	GCAATTAGCAAGACATCTTTTTAGAACACGCTGAATAAATTGAGGTT GCTGTGTAGGCTGGAGCTGCTTC	This study
norVW-mut-R	GGATAAGACGCGCCAGCGTCGCATCCGACATTTTAGGCACAGTAGC CCACCATATGAATATCCTCCTTAGTTCC	This study
norVW-verif-F	CACGCTGAATAAATTGAGGTTGC	This study
norVW-verif-R	GATAAGACGCGCCAGCGTC	This study
